# Larvivorous potentiality of *Puntius tetrazona* and *Hyphessobrycon rosaceus* against *Culex vishnui subgroup* in laboratory and field based bioassay

**DOI:** 10.1186/s13104-018-3902-8

**Published:** 2018-11-08

**Authors:** Mousumi Barik, Indranil Bhattacharjee, Anupam Ghosh, Goutam Chandra

**Affiliations:** 1 Mosquito Microbiology and Nanotechnology Research Units, Parasitology Laboratory, Department of Zoology, The University of Burdwan, Bardhaman, West Bengal India; 2 Department of Zoology, Dr. Bhupendra Nath Dutta Smriti Mahavidyalaya, Hatgobindapur, West Bengal India; 3 Department of Zoology, Bankura Christian College, Bankura, West Bengal India

**Keywords:** Biological control, *Puntius tetrazona*, *Hyphessobrycon rosaceus*, *Culex vishnui* subgroup

## Abstract

**Objectives:**

This study assessed the predatory potentiality of two unexplored fishes, *Puntius tetrazona* and *Hyphessobrycon rosaceus* on *Culex vishnui* subgroup larvae in order to utilize natural resources to diminish mosquito population. Larval feeding rate was evaluated in laboratory under varying prey density and volume of water. The experiment was extended to semi field condition.

**Results:**

*Puntius tetrazona* and *H. rosaceus* consumed from 66 to 600 and from 87 to 718 *Cx. vishnui* larvae respectively in laboratory condition at 10 prey density levels (100–1000 larvae) at an increment of 100 larvae at 2 l water volume. In semi field condition, a 78% reduction in larval density was observed at day 30 post introduction of *P. tetrazona*, whereas 91% reduction was noted on day 21 for *H. rosaceus* and in the subsequent samples no mosquito larvae were found in ditches. Withdrawal of predators from the ditches resulted gradual increase in larval density. Laboratory and semi field bioassay of both the species indicated their potentiality as efficient mosquito larval predator though *H. rosaceus* exhibited better performance than *P. tetrazona*. It is recommended to utilize these natural resources to diminish mosquito population in the countries of their native range.

**Electronic supplementary material:**

The online version of this article (10.1186/s13104-018-3902-8) contains supplementary material, which is available to authorized users.

## Introduction

Mosquitoes are blood sucking dipteran insects that act as vector of several important life threatening diseases, including malaria, lymphatic filariasis, chikungunya, dengue fever and Japanese encephalitis [1, 2]. To overcome these diseases, the reduction in the population size of mosquitoes is essential. The most effective means to control mosquitoes is the application of chemical insecticide. However, long term use of synthetic insecticides is harmful for human health and non target animal and arthropod populations and an important cause of environmental pollution. Also mosquitoes can become resistant to the synthetic chemicals after prolonged use. To overcome this problem, mosquito larvae management through biological control is preferred all over the world as an alternative and cost effective method [1–6]. Fishes are an important part of aquatic food chain and the mosquito larvae are significant part of their diet. In some of the previous studies, it was showed that the use of larvivorous fishes could represent a valid method for the biological control of mosquito immature stages in controlled and small natural habitats [7, 8].

The predation potentiality of a predator generally increases with the increase in prey density [9]. Except prey density, some other factors like species of prey item and its size, the species and size of predator, presence of vegetations in the aquatic habitat, water parameters and its quality, illumination intensity and prey capture or feeding method of the predator also influence the feeding efficacy of aquatic predators [1, 2, 5, 6, 10–15].

*Culex vishnui* subgroup is an important vector of Japanese encephalitis (JE) in India. JE is the most important viral encephalitis in Asia, especially in rural and suburban areas where rice culture and pig farming coexist [16]. A current estimate suggests that there are about 50,000 cases of JE globally having 15,000 death/year; in India [17]. This species breed in water with luxuriant vegetation mainly in paddy fields, shallow ditches and seasonal pools.

*Puntius tetrazona* (Bleeker, 1855), the tiger barb or Sumatra barb, belonging to the order cypriniformes and the family Cyprinidae is a tropical fish. The natural geographic range of this species extends throughout the Malay Peninsula, Sumatra and Borneo, with unsubstantiated sightings reported in Cambodia [18, 19]. Tiger barbs are also found in many other parts of Asia [20]. Tiger barbs prefer clear or turbid shallow waters of moderately flowing streams. They are brownish yellow in colour and characterized by presence of four vertical black stripes in body and have red fins and snout. *Hyphessobrycon rosaceus* Durbin, 1909, the rosy tetra belongs to the order Characiformes, family Characidae. It is a small species of fresh water fish from the South American countries of Guyana and Brazil [21, 22]. In the wild they come from areas with soft acid water, but the adults can tolerate natural or even slightly alkaline water. It is silver white in colour and characterized by red fins.

The present study was carried out to assess the predatory potential of *Puntius tetrazona* and *Hyphessobrycon rosaceus* on *Culex vishnui* subgroup larvae under laboratory conditions with reference to prey density and search area. The biocontrol efficacy of the two species was also assessed under semi field conditions.

## Main text

### Materials and methods

#### Collection of mosquito larvaes

Larvae of *Culex vishnui subgroup* (Diptera: Culicidae) were collected by fine netting from rice fields and different other aquatic bodies of Burdwan (23°16′N, 87°54′E). They were settled and reared for several generations in the laboratory. Third instar larvae were used for the experiments. The larvae grown in laboratory were fed with powdered dog biscuits [23].

#### Collection, maintenance and breeding of fishes and alternative prey organisms

The experiments were done in the Mosquito Research Unit, Parasitology Laboratory, Department of Zoology, The University of Burdwan, West Bengal, India. One pair of mature female and another pair of mature male of *P. tetrazona* (about 7.3 cm long) and *H. rosaceous* (about 3.9 cm long) were collected from the market. Males and females of each species were placed separately in four fiber glass water tanks (45.72 cm × 25.4 cm × 25.4 cm) containing 30 l of tap water (pH 7.2) and clusters of fine leaved plants with 1 kg of sand and 0.5 kg of pebbles at the bottom and maintained in the laboratory. The tanks were kept in dim light at room temperature on an average of 27.5 °C. Fishes were provided with required amount of fish food and *Tubifex* sludge worms. When the females were full of eggs and fatty enough and the males were brightly colourful, they were released in a spawning tank of same size and conditions containing slightly acidic water (pH 6.5). Spawning took place within 2 days and as soon as the eggs were found the fishes were withdrawn. After breeding the next generation stock population was used during bioassay experiment. Chironomid larvae (Order Diptera, Suborder Nematocera) and damselfly nymphs (Order Odonata, Suborder Zygoptera,) were collected from the wetlands located within the Burdwan University campus and settled in laboratory trays.

#### Evaluation of predation potential on *Cx. vishnui* larvae

The predatory potential of the fishes was assessed under varying volumes of water (2, 4 and 8 l) and varying densities of prey, i.e. third-instar larvae of *Cx. vishnui*. A single fish was released to predate on a specific prey density of 100–1000 with an increment of 100 larvae in each step (i.e., 10 density levels), in each water volume. For each water volume and prey density combination, three replications were done to assess the feeding trend of different individuals of the same fish species of the same size group. Control experiment was also set for different prey density and for different water volumes. The ranges of size and weight of *P. tetrazona* used in the experiment were 5–5.4 cm and 1.2–1.35 g. For *H. rosaceous* the ranges of size and weight used in the experiment were 3.4–3.6 cm and 0.91–0.96 g respectively. The experiments were carried out on seven different days, under similar conditions. After 7 days, data of altogether 630 observations were noted (3 levels of volume × 10 levels of prey density × 7 days × 3 replicates) per fish species. After each day the number of prey consumed was counted and the prey density was maintained constant by addition of equivalent numbers of prey. Multiple regression equation analysis was carried out considering predation by *Puntius tetrazona* and *Hyphessobrycon rosaceus* fish species (Y) as dependent variable on variable conditions of mosquito larvae density (X_1_) and water volume (X_2_). The data related to predation rate were subjected to four-way factorial ANOVA (p  < 0.01) [24].

#### Selection of mosquito larvae in presence of alternative prey

In a laboratory bioassay, a single individual of each predatory fish was allowed to predate on 200 specimens of each of three different preys, i.e. chironomid larvae, damselfly nymphs and *Cx. vishnui* subgroup kept in a single glass aquarium containing 2 l of water. After 24 h, the rate of consumption of each kind of prey was calculated for each fish. The experiments were repeated thrice for each predator fish species.

#### Evaluation of predatory efficiency in semi field condition

Predatory potentiality of the fishes was carried out in ditches located adjacent to rice fields of a village, Jotsadi, Burdwan, West Bengal, India. Average size of the ditches was 4 m long, 2 m wide and 0.3 m deep and drained excess rain water from rice fields. Twelve such ditches were selected which were disconnected from one another and sampled randomly for mosquito larvae to assess density [25] prior to introduction of the fishes. In the collected samples, the majority of the mosquito immature stages (larvae and pupae) were identified as *Cx. vishnui* subgroup. Few individuals were *Anopheles subpictus* sensu lato. In each of 5 ditches, 30 individuals of *P. tetrazona* were introduced. In each of other 5 ditches the same number of *H. rosaceous* were introduced. Two ditches were considered as control (without introduction of any fish). After introduction of the fishes, the average density of mosquito larvae in these ditches was estimated at 3-day interval for 30 days. After 30 days, fishes were removed from the ditches with fishing net and density of larvae was assessed every 3 days interval up to next 30 days. After laboratory and semi-field feeding experiments, all the fishes under study were released in a near-by pond in living condition in the University campus protected area. Data were subjected to t test analysis and distribution plot was drawn using XLSTAT software [24].

### Results

#### Predatory potential

The fishes consumed considerable numbers of third-instar *Cx. vishnui* subgroup though variation in feeding rate was noted depending upon prey density and the volume of water. The prey consumption ranged from 66 to 600 for *P. tetrazona* and from 87 to 718 for *H. rosaceus* in laboratory condition against third-instar mosquito larvae/day at 2 l water volume. With the increase in water volume (search area), the predation rate dwindled but the prey consumption increased linearly with the prey density irrespective of water volume (Additional file 1: Fig. S1a, b). The result from multiple regressions for predation of mosquito larvae by *P. tetrazona* was: Y = 70.794 X1 − 3.814 X2 + 11.994 and for *H. rosaeus* the corresponding equation was Y = 81.468 X1 − 0.488 X2 + 16.748. The results in both the cases suggested that the rate of predation (Y) by both predator species was positively correlated with prey density (X_1_) and inversely related to water volume (X_1_). The independent variables (*X*_1_ and *X*_2_) were strong predictors of feeding rate because for each fish species the multiple correlation coefficients (*R*^2^) were nearer to 1 (0.949 and 0.967 for *P. tetrazona* and *H. roseus* respectively).

The results of the four-way factorial ANOVA (Table 1) revealed significant differences in prey consumption among them. The daily predation rate at a specific prey density did not differ significantly for the 7-day experimental period for a particular predator species.

**Table 1:** Results from four-way factorial ANOVA of insect immature stages (*Culex vishnui* subgroup larvae) predation by *Puntius tetrazona* and *Hyphessobrycon rosaceus* larvivorous fishes at 10 different densities of insect immature stages and 3 different volumes of water, for 7 consecutive days

Source of variation	Sum of squares	df	Mean square	F	*p*-value
Fish species (FS)	1,548,544.11	1	1,548,544.11	25,221.24	0.01
Volume of water (V)	11,755.94	2	5877.96	95.74	0.01
Days (D)	280,636.60	6	46,772.76	761.79	0.01
Prey density (PD)	62,058,653.3	9	6,895,405.92	112,305.9	0.01
FS × V	11,272.94	2	5636.47	91.80	0.01
FS × D	29,339.23	6	4389.87	71.49	0.01
FS × PD	390,088.93	9	43,343.21	705.93	0.01
V × PD	43,452.57	18	2414.03	39.31	0.01
D × PD	47,654.710	54	882.495	14.373	0.01
FS × V × D	2119.108	12	176.592	2.876	0.01
FS × V × PD	54,595.719	18	3033.096	49.400	0.01
FS × D × PD	42,235.833	54	782.145	12.739	0.01
V × D × PD	45,216.105	108	418.668	6.819	0.01
FS × V × D × PD	30,559.114	108	282.955	4.609	0.01
Residual	51,574.67	840	61.39		
Total	64,647,699.00	1259			

#### Selection of mosquito larvae in presence of alternative prey

Results presented in Table 2 clearly show that mosquito larvae were the prey of choice among the three prey items viz. mosquito larvae, dragon fly nymphs and chironomid larvae.

**Table 2:** Number (mean ± S.E.) of *Culex vishnui* subgroup (*Cx. vishnui*) larvae, chironomid larvae and damselfly nymphs predated in a period of 24 h by *Puntius tetrazona* and *Hyphessobrycon rosaceus* fishes

Insect immature stages	*Puntius tetrazona*	*Hyphessobrycon rosaceus*
*Cx. vishnui* larvae	163.9 ± 7.8	174.3 ± 11.10
Chironomid larvae	55 ± 1.3	43 ± 2.9
Damselfly nymphs	48 ± 1.9	61 ± 2.4

#### Field evaluation of predatory efficiency

The result presented in Additional file 1: Fig. S2 indicates that the fish predators reduced the abundance of the mosquito larvae in all experimental ditches respect to the control ditches. Before introduction of the fishes, the mean density of mosquito larvae available in the ditches (control experiment) ranged between 252 and 275 (no. of larvae/dip). After the fishes were introduced, 91% reduction of mosquito larvae was observed on day 21 for *H. rosaceus* and 78% reduction in the density was observed for *P. tetrazona* on day 30. Withdrawal of the fishes from the ditches facilitated gradual increase in larval population in the next 30 days. The mean densities of mosquito larvae was significantly reduced in presence of the fishes in comparison to the control ditches without fishes (for *P. tetrazona*: t = 10.933, for *H. rosaceus*: t = 12.673; df = 53, p < 0.0001), as shown in Additional file 1: Fig. S3.

### Discussion

This is the first report on predation of two fish species, *P. tetrazona* and *H. rosaceus*, on mosquito larvae. The present study shows negative correlation between quantity of water and feeding efficiency. This is consistent with data reported in some previous studies on other fish species [1, 2, 5, 6]. The foraging behavior of the fishes altered with the increase in search area; as a result consumption of the mosquito larvae decreased [1, 2, 5, 6]. After 30 days of introduction of fishes in the treated ditches larval density greatly reduced as also reported by other authors [1, 5, 6, 26]. Gradual increase in larval density on withdrawal of predator fishes from the ditches in next 30 days further confirms this result. Therefore, these fishes could be used as useful tools for biological control of mosquitoes in different kinds of fresh water habitats like ponds, pools, rice fields etc. Considering the biocontrol potential of *P. tetrazona* and *H. rosaceus* observed in this study, these two fish species could be used as a mean for the control of mosquitoes, especially *Cx. vishnui* subgroup. Nevertheless, further studies are needed on the effects on aquatic communities of the use of these fishes in the field, in order to fully evaluate their overall impact on biodiversity and benefits in the control of mosquito populations.

## Limitations

The experiment was not conducted in field condition.

## Additional file

**Additional file 1: Figure S1a.** Number of third-instar *Culex vishnui* subgroup (*Cx. vishnui*) larvae per liter of water consumed by *Puntius tetrazona* under laboratory conditions. For each density of mosquito larvae, the values represented by the bars are means (± SE) of three repetitions in a 7-day observation period. **Figure S1b.** Number of third-instar *Culex vishnui* subgroup (*Cx. vishnui*) larvae per liter of water consumed by *Hyphessobrycon rosaceus* under laboratory conditions. For each density of mosquito larvae, the values represented by the bars are means (± SE) of three repetitions in a 7-day observation period. **Figure S2.** Mean density of mosquito immature stages/day observed in a period of 60 days in ditches containing *Puntius tetrazona* or *Hyphessobrycon rosaceus* fishes (first 30 days with the fishes and next 30 days after withdrawal of fishes) respect to control ditches without fishes. **Figure S3.** Distribution plots of t-test (two tailed paired samples).

## Abbreviations

ANOVA: analysis of variance
JE: Japanese encephalitis
