# Supplementary material for: Larvivorous potentiality of Puntius tetrazona and Hyphessobrycon rosaceus against Culex vishnui subgroup in laboratory and field based bioassay
Source: BMC Res Notes. 2018 Nov 8;11:804. doi: 10.1186/s13104-018-3902-8 (PMC6225676; doi:10.1186/s13104-018-3902-8)
Supplement: Supplementary file 1 — Additional file 1: Figure S1a. Number of third-instar Culex vishnui subgroup (Cx. vishnui) larvae per liter of water consumed by Puntius tetrazona under laboratory conditions. For each density of mosquito larvae, the values represented by the bars are means (± SE) of three repetitions in a 7-day observation period. Figure S1b. Number of third-instar Culex vishnui subgroup (Cx. vishnui) larvae per liter of water consumed by Hyphessobrycon rosaceus under laboratory conditions. For each density of mosquito larvae, the values represented by the bars are means (± SE) of three repetitions in a 7-day observation period. Figure S2. Mean density of mosquito immature stages/day observed in a period of 60 days in ditches containing Puntius tetrazona or Hyphessobrycon rosaceus fishes (first 30 days with the fishes and next 30 days after withdrawal of fishes) respect to control ditches without fishes. Figure S3. Distribution plots of t-test (two tailed paired samples). [file 13104_2018_3902_MOESM1_ESM.docx]

**Figure Legends:**

**Figure Title**

Figure S1a. Number of third-instar *Culex vishnui* subgroup (*Cx. vishnui*) larvae per liter of water consumed by *Puntius tetrazona* under laboratory conditions. For each density of mosquito larvae, the values represented by the bars are means (± SE) of three repetitions in a 7-day observation period.

| Mosquito larvae |
| --- |

Figure S1b. Number of third-instar *Culex vishnui* subgroup (*Cx. vishnui*) larvae per liter of water consumed by *Hyphessobrycon rosaceus* under laboratory conditions. For each density of mosquito larvae, the values represented by the bars are means (± SE) of three repetitions in a 7-day observation period.

|  2 liters  4 liters 8 liters |
| --- |

| Mosquito larvae |
| --- |

Figure S2. Mean density of mosquito immature stages/day observed in a period of 60 days in ditches containing *Puntius tetrazona* or *Hyphessobrycon rosaceus* fishes (first 30 days with the fishes and next 30 days after withdrawal of fishes) respect to control ditches without fishes.

Figure S3. Distribution plots of t- test (Two tailed paired samples)

| a) *t- test* (paired) plot of control experiment vs experiment with *P. tetrazona* fish | b*) t- test* (paired) plot of control experiment vs experiment with *H. rosaceus* fish |
| --- | --- |
|  |  |
| \| Difference \| 108.333 \| \| --- \| --- \| \| t (Observed value) \| 10.933 \| \| \|t\| (Critical value) \| 2.006 \| \| DF \| 53 \| \| p-value (Two-tailed) \| < 0.0001 \| \| alpha \| 0.05 \|   **Test Interpretation:**  H0: The difference between the means is equal to 0.  Ha: The difference between the means is different from 0.  As the computed p-value is lower than the significance level alpha=0.05, the null hypothesis H0 is rejected, and alternative hypothesis Ha is accepted. | \|  \|  \|  \|  \|  \| \| --- \| --- \| --- \| --- \| --- \|  \| Difference \| 166.204 \| \| --- \| --- \| \| t (Observed value) \| 12.673 \| \| \|t\| (Critical value) \| 2.006 \| \| DF \| 53 \| \| p-value (Two-tailed) \| < 0.0001 \| \| alpha \| 0.05 \|   **Test Interpretation:**  H0: The difference between the means is equal to 0.  Ha: The difference between the means is different from 0.  As the computed p-value is lower than the significance level alpha=0.05, the null hypothesis H0 is rejected, and alternative hypothesis Ha is accepted. |
